# Enhancing *Vibrio vulnificus* infection diagnosis for negative culture patients with metagenomic next-generation sequencing

**DOI:** 10.3389/fcimb.2023.1210919

**Published:** 2023-11-16

**Authors:** Xinghua Li, Chengzhuo Wang, Zhaowang Guo, Tongyang Xiao, Yuxin Ji, Yongguang Ma, Meiyi Li, Jinyu Xia, Xi Liu

**Affiliations:** ^1^ Department of Infectious Diseases, The Fifth Affiliated Hospital of Sun Yet-sun University, Zhuhai, China; ^2^ Clinical Laboratory, The Fifth Affiliated Hospital of Sun Yat-sen University, Zhuhai, China; ^3^ Department of Orthopaedics, The Fifth Affiliated Hospital of Sun Yat-sen University, Zhuhai, China

**Keywords:** Vibrio vulnificus, metagenomic next-generation sequencing, culture, necrotizing fasciitis, surgery

## Abstract

**Objective:**

To evaluate the diagnostic value of metagenomic next-generation sequencing (mNGS) in *Vibrio vulnificus* (*V. vulnificus*) infection.

**Methods:**

A retrospective analysis of patients with *V. vulnificus* infection at the Fifth Affiliated Hospital of Sun Yat-Sen University from January 1, 2020 to April 23, 2023 was conducted. 14 enrolled patients were diagnosed by culture or mNGS. The corresponding medical records were reviewed, and the clinical data analyzed included demographics, epidemiology laboratory findings, physical examination, symptoms at presentation, antibiotic and surgical treatment, and outcome.

**Results:**

In this study, 78.6% (11/14) patients had a history of marine trauma (including fish stab, shrimp stab, crab splints and fish hook wounds), 7.1% (1/14) had eaten seafood, and the remaining 14.3% (2/14) had no definite cause. Isolation of *V. vulnificus* from clinical samples including blood, tissue, fester and secreta. 9 cases were positive for culture, 5 cases were detected synchronously by mNGS and got positive for *V. vulnificus*. 85.7% (12/14) cases accepted surgical treatment, with 1 patient suffering finger amputated. 14 enrolled patients received appropriate antibiotic therapy, and all of them had recovered and discharged. 9 strains *V. vulnificus* isolated in this study were sensitive to most beta-lactam antibiotics, aminoglycosides, quinolones, etc.

**Conclusion:**

*Vibrio vulnificus* infection is a common water-exposed disease in Zhuhai, which requires identification of a number of pathogens. Of severe infections with unknown pathogen, mNGS can be used simultaneously, and the potential to detect multiple pathogens is of great help in guiding treatment.

## 1 Introduction

*Vibrio vulnificus* (*V. vulnificus*) is a gram-negative aquatic bacterium first isolated by the United States in1967 and an important opportunistic pathogen. Human infections associated with this bacterium originate from two distinct sources: consumption of seafood (primary septicemias) or exposure to seawater or seafood products (wound infections) (Hollis et al., 1976; Blake et al., 1979; Johnston et al., 1986).As a serious public health concern around the world, *V. vulnificus* has a high mortality 50% in USA (Jones and Oliver, 2009), 48.9% in Korea (Kim et al., 2022), almost 31.6% in China (Hsueh et al., 2004; Hong et al., 2014; Cai et al., 2021). What needs to be pointed out is mortality of *V. vulnificus* was significantly elevated with delayed initiation of anti-infective treatment (Klontz et al., 1988).

For patients with *V. vulnificus* infection, the disease progresses rapidly. It is of great value to identify the pathogenic pathogen and adjust the empirical treatment plan in time for the prognosis of the disease.

Presently, the gold standard for the etiological examination of *V. vulnificus* is still the traditional microbial culture technique, including blood, blister and cerebrospinal fluid. However, traditional culture methods are time-consuming, and the data show that the overall positive rate remains at a relatively low level of about 30% to 40% (Burillo and Bouza, 2014). After the application of antibacterial drugs, the positive rate of blood culture is significantly decreased (tissue and tissue secretion culture is higher), which often brings difficulties for clinical diagnosis.

Metagenomic next generation sequencing (mNGS) is a non-targeted and broad-spectrum pathogen screening technology. In recent years, more and more studies have shown that mNGS pathogen detection plays a key role in the field of clinical severe infection (Chiu and Miller, 2019), especially when the severe infection was caused by rare pathogens (Bullman et al., 2017; Miao, 2018).

Thus, to evaluate the pathogen- detecting performance of mNGS and traditional culture in *V. vulnificus* infectious diseases, we retrospectively analyzed the characteristics of 14 V*. vulnificus* infections diagnosed by mNGS and culture from the Fifth Affiliated Hospital of Sun Yat-sen University from January 1, 2020 to April 23, 2023.

## 2 Materials and methods

### 2.1 Case screening and inclusion

We retrospectively reviewed the medical records of patients infected with *V. vulnificus* who were admitted to the Fifth Affiliated Hospital of Sun Yat-Sen University, Zhuhai, China, from January 1, 2020 to April 23, 2023. The study hospital is a university-affiliated and tertiary hospital with 2300 beds, with an annual discharge of 67,100 patients and an outpatient volume of approximately 1.13 million. It is one of the largest tertiary hospitals in Guangdong Province.

Medical records were reviewed for clinical information, including demographics, epidemiology laboratory findings, physical examination, symptoms at presentation, antibiotic and surgical treatment, and outcome.

### 2.2 Culturing procedure and disk sensitivity tests

Isolation of *V. vulnificus* from clinical samples including blood, tissue, fester and secreta. *V. vulnificus* in culture-positive samples were identified by conventional phenotypic identification methods and Bruker MALDI-TOF MS systems (Bruker Daltonics, Billerica, MA, USA) (Barberis et al., 2017).

#### 2.2.1 Conventional phenotypic identification methods

All isolates were identified by conventional phenotypic methods. Phenotypic identification of colonies included morphology, Gram staining, catalase activity, lipophily for Corynebacterium spp. and biochemical methods using the algorithm previously described by Funke et al. In parallel with conventional biochemical methods, molecular identification was performed.

Molecular identification was used as the gold standard method to compare the results obtained by MALDI TOF MS and conventional phenotypic methods. 16S rRNA gene sequencing was carried out for characterization of all isolates. In Corynebac terium spp. identification, rpoB gene was used as a secondary gene target when 16S rRNA gene did not allow correct identification to species level. Sequencing of the PCR products were performed on both DNA strands using ABIPrism 3100 BioAnalyzer equipment at Macrogen Inc. sequencing facility, South Korea, sequencing facility. The sequences were analyzed using the BLAST v2.0 software (http://www.ncbi.nlm.nih.gov/BLAST/). A $99,0% (16S rRNA gene) and $95,0% (rpoB gene) similarity cut-off was required for species identification.

#### 2.2.2 MALDI-TOF mass spectrometry

The clinical strains from the culture collection were subcultured on Columbia agar containing 5% sheep blood (Laboratorios Britania, Argentina) at 37uC with 5% CO2 for 24 to 48 h for MALDI-TOF MS measurement. Bacterial isolates were identified by the direct colony on plate extraction method [10]. MALDI-TOF target plates were inoculated into the spots by picking a freshly grown overnight colony and overlaid with 1 ml of 70% formic acid (Sigma-Aldrich). Each spot was allowed to dry and subsequently overlaid with 1 ml of matrix (a-cyano-4- hydroxycinnamic acid)

Mass spectra were acquired using the MALDI-TOF MS spectrometer in a linear positive mode (Microflex, Bruker Daltonics). The bacterial test standard (BTS, Bruker) was used for instrument calibration. Mass spectra were analyzed in a m/z range of 2,000 to 20,000. The MALDI Biotyper library version 3.0 and MALDI Biotyper software version 3.1 were used for bacterial identification. Based on previous studies cut-off scores for identification were: $1,5 for genus level, $1,7 for species-level. A score, 1,5 was considered as resulting in no reliable identification. A minimum difference of 10% between the top and next closest score was required for a different genus or species.

#### 2.2.3 Disk sensitivity tests

The culture plates were incubated at 37°C for 24 h to impart a suitable environment for the cultivation of the microorganisms, following which the culture plates were transferred to a laminar air flow chamber to prevent contamination and maintain an aseptic environment. Five wells of standardized diameter were prepared with help of Durham’s tube, systematically placed equidistantly from each other to avoid conjoining of subsequent zones of surrounding wells upon each other. Three wells were designated for the test materials and two for control in which normal saline and distilled water was placed (Ahmed et al., 2022).

Minimum inhibitory concentration and minimum bactericidal concentration values were regulated by performing triplicate test tube dilution methods for the test samples. The test solution used was <0.1% concentration which was regarded as the lowest concentration allowed to cultivate on the medium.

Culture plates were incubated at 37°C temperature under required humidity and gaseous environment for 24 h, and subsequently 48 h.

The inhibitory zone is contemplated to be the shortest measurement of length from the exterior circumference of the circular well to the preliminary site of microbial growth. Regions depicting the suppression of microbial expansion around the circular well depicts the zone of inhibition. Measurements of zones of inhibition were recorded with help of an electronic caliper and readings were subjected to statistical analysis.

The criteria for judging Vibrio vulnificus were based on the MIC interpretation criteria of CLSI M100 Ed33 Gram-negative Vibrio.

### 2.3 mNGS diagnostic method

#### 2.3.1 Specimen collection and laboratory procedures

Samples taken from patients include blood and tissue secretions. They were stored in dry ice and sent to BGI Shenzhen (collection, preservation and transportation of mNGS specimens were strictly in accordance with the company’s standardized specifications) for mNGS detection. The progress of mNGS, data analysis and criteria for a positive result was the same as the previously described a detailed description follows (Liu et al., 2021).

#### 2.3.2 Sample processing and DNA extraction

A 1.5 mL microcentrifuge tube containing 0.5 mL of sample and 1 g of 0.5 mm glass beads was connected to the horizontal platform of the vortex mixer and stirred vigorously at 2800-3200 RPM for 30 minutes. A 0.3 mL sample was then separated into a new 1.5 mL microcentrifuge tube and the TIANamp Micro DNA kit (DP316, TIANGEN BIOTECH) was used to extract nucleic acids according to the manufacturer’s instructions.

#### 2.3.3 Construction of DNA libraries and sequencing

A DNA library was constructed by DNA fragmentation, end-repair, adaptor ligation, and polymerase chain reaction (PCR) amplification. The Agilent 2100 (Agilent Technologies, Santa Clara, CA) and Qubit 2.0 (Invitrogen, USA) were used for the library quality control. The qualified double-stranded DNA library was converted into a single-stranded circular DNA library through DNA denaturation and circularization. Rolling circle amplification (RCA) was used to generate DNA nanoballs (DNBs) from single-stranded circular DNA. DNBs were qualified using Qubit 2.0 and then loaded into the flow cell, and single-end 50 cycles sequencing was performed on the BGISEQ-50 platform.

#### 2.3.4 Bioinformatic analysis

The low-quality reads and short (length <35 bp) reads were deleted from the raw data using in-house software. The remaining high-quality data were mapped to the human reference genome (hg19) using Burrows-Wheeler Alignment. These reads in the alignment were removed as the human host sequence. After the removal of low-complexity reads, the remaining data were simultaneously aligned to Microbial Genome Databases (including viruses, bacteria, fungi, and parasites) for classification. The databases were downloaded from NCBI (ftp://ftp.ncbi.nlm.nih.gov/genomes/) which contains 4061 whole-genome sequences of virus, 2473 bacterial genomes or scaffolds, 199 fungi, and 135 parasites related to human diseases. The number of unique alignments reads was calculated. The coverage ratio and depth of each microorganism were calculated using BED Tools.

#### 2.3.5 The Interpretation of Metagenomic Analysis

*V. vulnificus* was considered positive when at least 1 read was mapped to either the species or genus level due to the difficulty of DNA extraction and a low possibility for contamination.

### 2.4 Statistical analyses

Continuous data was reported as mean (standard deviation). Independent sample t-test was implemented for comparison of normally distributed continuous variables between two groups. They were performed using SPSS 22.0 software. *P* values ≤ 0.05 (*P* ≤ 0.05**) were considered significant and all tests were 2-tailed.

## 3 Result

### 3.1 Clinical features of 14 V*. vulnificus* infections

14 microbiology-proven patients of *V. vulnificus* infection (3 females and 11 males) with different underlying disease were included in the study, the average age was 58.86 ( ± 10.63) years old. Among the included patients, their occupations varied, but 78.6% (11/14) had a history of marine trauma (including fish stab, shrimp stab, crab splints and fish hook wounds), 7.1% (1/14) had eaten seafood, and the remaining 14.3% (2/14) had no definite cause. Except for the 3 patients with no clear cause or no clear contact time, the remaining patients who had a clear history of contact developed the corresponding symptoms of *V. vulnificus* infection within 24 hours. These related symptoms include fever, chill, shock, sore throat, fatigue, headache and vomit, which is concretely shown in **Table 1**, and all but one patient came to the hospital within 1 day after the onset of symptoms. All patients had infection-like inflammatory reactions (redness, swelling, heat, pain) of corresponding limbs. Tissues, secretions and pustular fluid from lesion limbs were collected for pathogen detection.

**Table 1:** Basic features of 14 V*. vulnificus* infections.

No.	Age	Gender	Occupation	Exposure route	Underlying disease	Time from exposure to onset (hours)	Time from exposure to admission(hours)	Symptoms	Inflammatory site
1	72	female	retirement	eat seafood	HAV infection、Bilharziasis、CKD、CRF、AA、Gall-stone	UNK	UNK	fever, chill, sore throat	left forearm
2	67	male	retirement	fish stab	Gastric perforation、Valve replacement 、MI	9	12	fever, chill, vomit	left forearm
3	63	male	UNK	fish stab	UNK	2	17	fever, shock	left lower limb
4	64	male	peasant	fish stab	HBV infection、rapid atrial fibrillation、severe tricuspid regurgitation	in 24	in 48	fever, shock	right hand、 right hip
5	64	male	UNK	shrimp stab	UNK	in 24	in 24	fever, chill, vomit	right hand
6	40	male	peasant	fish stab	UNK	in 24	in 72	fever	right foot
7	68	male	UNK	UNK	Gouty arthritis、Osteonecrosis of the femoral head、gout	UNK	UNK	Null	right lower limb
8	48	male	peasant	UNK	Diabetes、HBV infection、Fatty liver、Cushing-like syndrome、Jock itch	UNK	UNK	shock	right upper limb
9	66	male	retirement	crab splints	Hypertension	in 24	in 24	shock, fatigue	left thumb
10	59	male	worker	fish stab	Hypertension	24	in 48	fever	right forefinger
11	43	male	UNK	fishhook stab	HBV infection	in 24	in 24	Null	right hand
12	58	female	retirement	fish stab	UNK	in 24	in 24	fever, chill, fatigue	left toe
13	69	male	fishmonger	fish stab	CKD、atrial fibrillation	in 24	in 24	fever, chill, fatigue, headache	right opisthenar
14	43	female	UNK	fish stab	Null	in 24	in 24	Null	right hand

UNK, Unknown.

**Figure 1:**
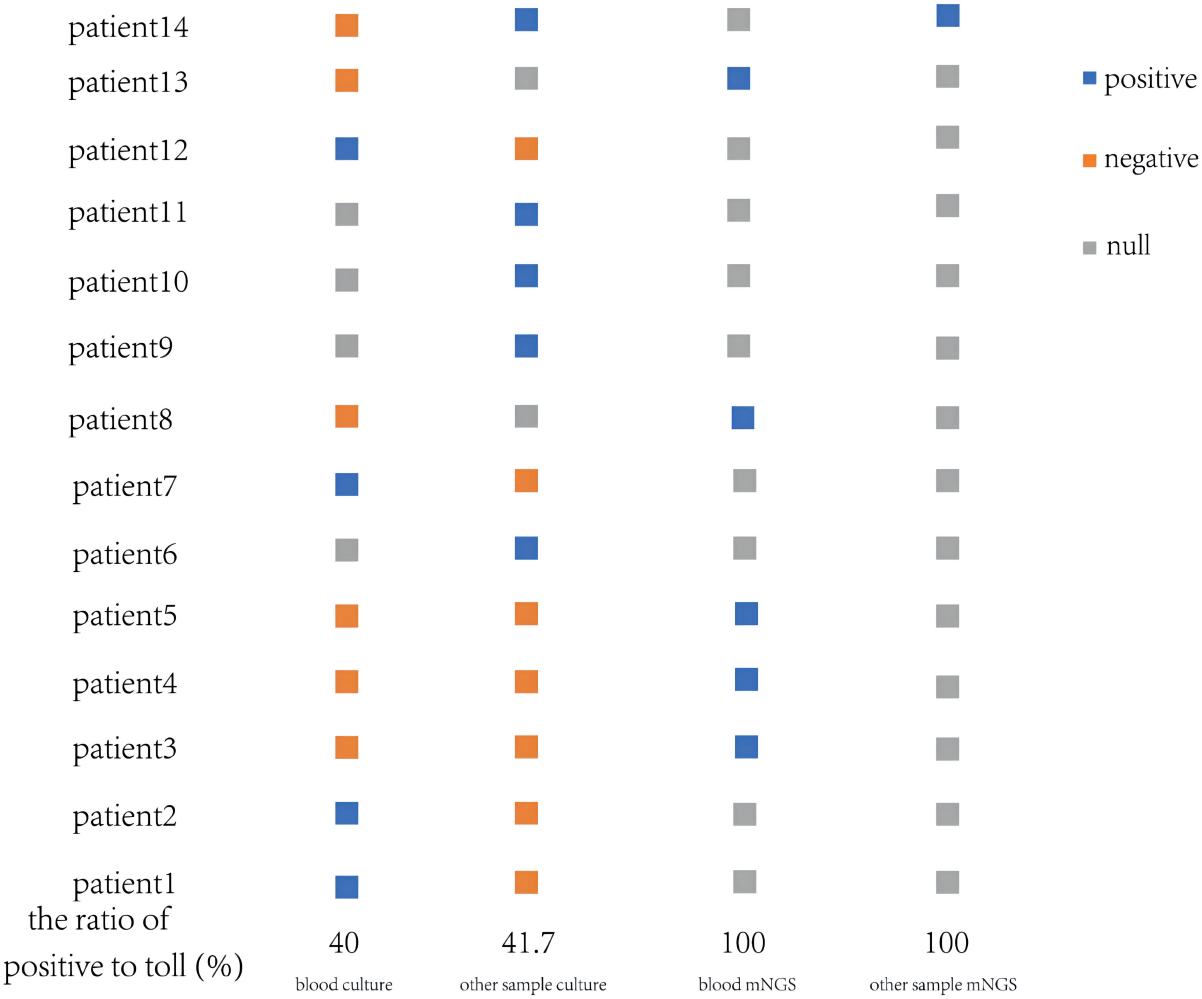
*V. vulnificus* detection by culture and mNGS from blood and other samples of 14 patients.

The corresponding samples of these patients were cultured respectively, two or more samples from 8 patients were collected for culture. Their *V. vulnificus* culture results can be seen in **Figure 1**, 4 out of 10 blood samples were positive, 5 out of 12 other samples were positive. With the exception of one patient who was given an etiological culture and mNGS upon admission, eight of the remaining patients were confirmed to be infected with *V. vulnificus* after the etiological culture, while the other 5 patients with negative samples were tested for the next step of etiological examination-mNGS. Fortunately, *V. vulnificus* was detected in all 5 blood samples and 1 other sample through mNGS, providing strong evidence for precisely diagnosis. Besides, we could easily compare the difference between pathogen culture and mNGS. In the following test methods, blood culture positive rate was 40% (4/10), the other sample positive rate was 41.7% (5/12), and mNGS positive rate was 100%. Obviously, among these methods, mNGS exhibited excellent detection rates, while the detection rates of blood were relatively low.

Similarly, some laboratory biochemical indicators presented significant changes (concrete value were shown in **Table 2**). 78.6% (11/14) patients had elevated white blood cells, all patients had increased neutrophil percentages, procalcitonin and C-reactive protein. 35.7% (5/14) had abnormal liver function and 42.3% (6/14) had abnormal clotting function. There was no significant differences in these laboratory indicators between the culture-positive and mNGS-positive patients.

**Table 2:** Laboratory findings of *Vibrio vulnificus* infections.

	Total N=14	Culture-positive N =9	mNGS-positive N =6	*P* value
WBC	17.57 ( ± 9.19)	17.46 ( ± 7.58)	19.77 ( ± 12.33)	.658
HGB	130.14 ( ± 25.88)	120.22 ( ± 25.31)	144 ( ± 18.03)	.069
PLT	154.86 ( ± 61.25)	154.78 ( ± 72.80)	165 ( ± 44.02)	.753
GRA%	89.69 ( ± 4.62)	89.97 ( ± 5.01)	90.58 ( ± 5.13)	.821
PCT	14.08 ( ± 16.25)	16.47 ( ± 19.76)	8.18 ( ± 6.95)	.273
ALT	40.41 ( ± 32.88)	42.14 ( ± 36.02)	37.48 ( ± 27.68)	.797
AST	40.45 ( ± 33.67)	35.81 ( ± 24.41)	44.7 ( ± 43.09)	.632
Tbil	16.72 ( ± 11.86)	19.45 ( ± 14.51)	12.15 ( ± 3.46)	.207
TP	61.92 ( ± 8.33)	64.98 ( ± 8.01)	59.6 ( ± 8.79)	.256
Alb	35.14 ( ± 5.80)	37.06 ( ± 6.07)	34.40 ( ± 6.83)	.456
Cr	128.3 ( ± 58.38)	111.5 ( ± 45.51)	136.57 ( ± 77.82)	.465
BUN	10.34 ( ± 4.70)	11.48 ( ± 5.69)	8.36 ( ± 0.94)	.202
PT	15.76 ( ± 7.74)	16.74 ( ± 9.52)	13.52 ( ± 2.69)	.438
INR	1.43 ( ± 0.72)	1.52 ( ± 0.88)	1.23 ( ± 0.27)	.451
HDL	1.32 ( ± 0.72)	1.41 ( ± 1.10)	1.25 ( ± 0.46)	.799
LDL	1.30 ( ± 0.88)	1.08 ( ± 0.40)	1.47 ( ± 1.16)	.612
TG	1.78 ( ± 2.11)	2.45 ( ± 3.41)	1.27 ( ± 0.61)	.610
TC	3.64 ( ± 1.24)	3.81 ( ± 0.96)	3.51 ( ± 1.37)	.760
ESR	40 ( ± 38.41)	43.86 ( ± 41.4)	24 ( ± 32.83)	.435
Lac	4.01 ( ± 2.85)	Null	4.01 ( ± 2.85)	Null
CK-MB	287.53 ( ± 364.54)	121.63 ( ± 105.58)	468.13 ( ± 450.02)	.120
LDH	215.06 ( ± 88.44)	182.72 ( ± 61.92)	260.19 ( ± 96.52)	.092
CRP	149.16 ( ± 92.68)	139.33 ( ± 104.00)	136.35 ( ± 99.15)	.959

All results are expressed as mean (standard deviation).

WBC, White blood cell; HGB, Hemoglobin; PLT, Platelet; GRA%, Granulocyte%; PCT, Procalcitonin; ALT, Alanine transaminase; AST, Aspartate transaminase; TBil, Total bilirubin; TP, Total protein; Alb , Albumin; Cr, Creatinine; BUN, Blood urea nitrogen; PT, Prothrombin time; INR, International normalized ratio; HDL, High density lipoprotein; LDL, Low density lipoprotein; TG, Triglyceride; TC, Total cholesterol; ESR, Erythrocyte sedimentation rate; Lac, Lactic acid; CK-MB , Creatine kinase-MB; LDH, Lactate dehydrogenase; CRP, C-reactive protein.

### 3.2 Therapeutic regimens and outcomes of *V. vulnificus* infections

Among all the 14 patients, surgical treatments were performed in 12 patients, including 10 cases of skin incision and Vacuum Sealing Drainage (VSD) and 2 cases of simple skin incision. Besides, all patients were accepted standard *V. vulnificus* anti-infective therapy (third-generation cephalosporin combined with quinolone). After surgical and medical treatments, 13 of the infected patients, eventually recovered, but unfortunately one of them, a 64-year-old male who was injured by shrimp, had his fourth finger amputated. Of 13 discharged patients, there were no significant differences in the length and cost of hospitalization between culture-positive and mNGS-positive patients. The length of hospitalization was 22.1 ( ± 18.9) days of culture-positive patients, and 28.6 ( ± 19.2) days of mNGS-positive patients (*P*=0.478). The total cost of hospitalization was 47269 ( ± 41313) yuan of culture-positive patients, and 98308 ( ± 61579) yuan of mNGS-positive patients (*P*=0.099) (**Table 3**).

**Table 3:** Therapeutic regimens and outcomes of *V. vulnificus* infections.

No.	Antibacterial agents	Surgical therapy	Outcomes	Length of hospital stay (days)	Cost of hospital stay (yuan)
1	Cefoperazone-sulbactam +Levofloxacin	Skin incision and VSD drainage	Recovery	33	90287
2	Cefoperazone sulbactam +Moxifloxacin	Skin incision and drainage	Recovery	10	19654
3	Cefoperazone-sulbactam +Moxifloxacin	Skin incision and VSD drainage	Recovery	36	116608
4	Cefoperazone-sulbactam +Ciprofloxacin	Skin incision and VSD drainage	Recovery	53	175675
5	Cefoperazone-sulbactam +Moxifloxacin	Skin incision and VSD drainage	Right finger excision	36	123001
6	Cefoperazone-sulbactam +Moxifloxacin	Skin incision and VSD drainage	Recovery	31	67216
7	Piperacillin -tazobactam +Moxifloxacin	Skin incision and VSD drainage	Recovery	24	113055
8	Piperacillin- tazobactam +Moxifloxacin	Null	Recovery	10	60423
9	Levofloxacin	Skin incision	Recovery	11	7531
10	Ceftazidime+Levofloxacin	Skin incision	Recovery	5	5631
11	Ceftriaxone+Levofloxacin	Skin incision and VSD drainage	Recovery	21	13930
12	Piperacillin- tazobactam	Skin incision and VSD drainage	Recovery	42	60850
13	Cefoperazone-sulbactam +Levofloxacin	Null	Recovery	8	15833
14	Ceftriaxone+ Levofloxacin	Skin incision and VSD drainage	Recovery	Inhosptial	Inhospital

**Figure 2:**
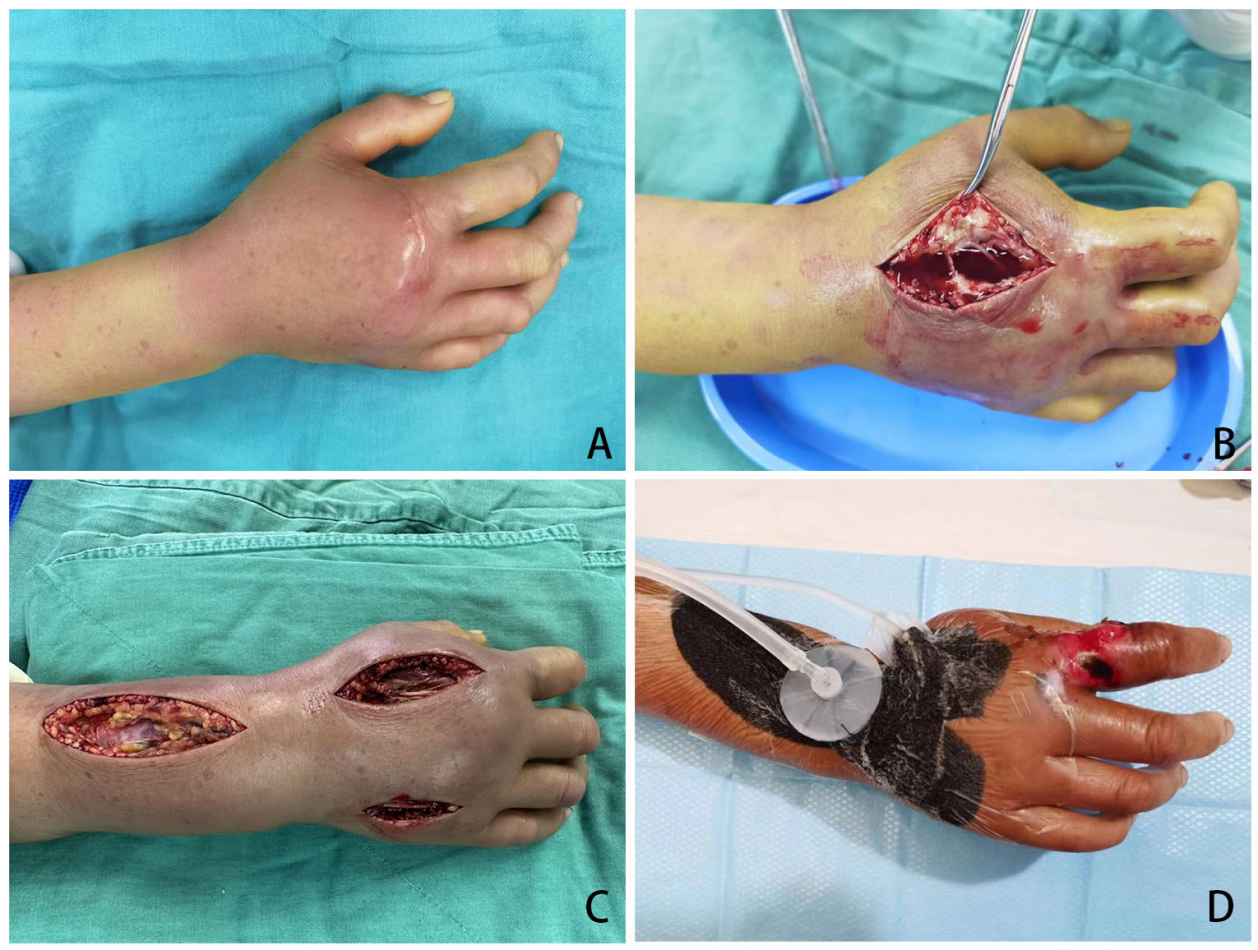
Necrotizing fasciitis of right hand of patient No. 14 after fish sting. **(A)** Pre-operation, 8 hours after fish sting; **(B)** Open decompression was performed at the position with the highest pressure on the back of the hand; **(C)** Three incisions were made on the hand and forearm for decompression; **(D)** Incision decompression combine with Vacuum Sealing Drainage.

A 43-year-old women (patient No. 14) presented to the emergency department on April 17, 2023, with 8 hours history of swelling pain in her right hand after fish sting. At the time of presentation, hemorrhagic bullae measuring 4 by 5 cm had developed on the back (or dorsal region) of her right hand. Decompression and vacuum sealing drainage was performed urgently, and *Vibrio vulnificus* was isolated from the bullae (**Figure 2**).

### 3.3 Results of disk sensitivity tests for 9 *Vibrio vulnificus* isolated from 9 patients

9 strains *V. vulnificus* isolated in this study were sensitive to most beta-lactam antibiotics, including Piperacillin, Ampicillin, Ceftazidime, Ciprofloxacin, Cefuroxime, Cefepime, Imipenem, Meropenem. 1 strain was resistant to Cefazolin, 3 strain were resistant to Cefoxitin. These *V. vulnificus* were also sensitive to aminoglycosides, quinolones, and some new synthetic antibiotics (**Table 4**).

**Table 4:** Results of disk sensitivity tests for 9 strains *V. vulnificus* isolated in this study.

Antibacterial agents	No. of sensitive isolates/no. of tested (%)
Ampicillin	2/2 (100)
Ampicillin/Sulbactam	2/2 (100)
Amoxicillin clavulanate potassium	2/2 (100)
Piperacillin	2/2 (100)
Piperacillin/Tazobactam	7/7 (100)
Ceftazidime	9/9 (100)
Ciprofloxacin	7/7 (100)
Cefuroxime	5/5 (100)
Cefazolin	0/1 (0)
Cefepime	8/8 (100)
Cefoperazone-sulbactam	7/7 (100)
Cefoxitin	0/3 (0)
Trimethoprim - sulfamethoxazole	2/2 (100)
Gentamicin	4/4 (100)
Imipenem	8/8 (100)
Levofloxacin	9/9 (100)
Meropenem	7/7 (100)
Amikacin	9/9 (100)
Cotrimoxazole	7/7 (100)
Tigecycline	6/6 (100)

## 4 Discussion

*V. vulnificus* occurs naturally in temperate estuarine and coastal waters worldwide, but is most frequently isolated when water temperatures are above 20°C and salinities are between 0.5-2.5% (Barberis et al., 2017). As a coastal city, Zhuhai is rich in fishery resources, and seafood is the popular food. Meanwhile, both selling and processing of the seafood increase the risk of aquatic bacterial exposure. In addition to primary sepsis, *V. vulnificus* often causes skin and soft tissue infection, mainly manifesting as redness, blisters, necrotic fasciitis, etc., with rapid progress and requiring surgical debridement or amputation (Ahmed et al., 2022). In the Oysters samples in Guangdong (Guangzhou, Jiangmen, Zhuhai), the carriage rate of *V. vulnificus* reached 25% (Liu et al., 2021).

In our study, 11/14 (78.6%) patients diagnosed with *V. vulnificus* were infected by exposed wounds, obviously higher than infected through dietary route. This may be related to the large number of fishery workers in Zhuhai. Fish and shrimp bites, fish hook scratches, and seawater immersion wounds are all wounds related to seawater exposure, and a variety of microorganisms can lead to soft tissue infection after exposure (Mead et al., 1999; OLIVER, 2005). In addition to *V. vulnificus*, there are many other species of bacteria that can infect people through marine wounds, and they can also cause superficial soft tissue and invasive systemic infections, including *Aeromonas hydrophila* (Yu et al., 2021), *Streptococcus iniae* (Diaz, 2014), *Mycobacterium marinum* (Diaz and Lopez, 2015) and *Vibrio vulnificus*. Early identification of the bacteria associated with wounds is particularly critical for adopting timely and appropriate treatment options.

Traditional laboratory examination of pathogenic microbiology mainly consists of staining, culture, biochemical identification, etc. Direct smear staining microscopy of specimens and inoculation on the medium for isolation and culture are common methods for the etiological diagnosis of bacterial or fungal infectious diseases. In recent years, molecular tests (typically PCR) and matrix-assisted laser desorption/ionization-time of flight mass spectrometry(MALDI-TOF MS)have also been widely used in etiological diagnosis (Gold and Salit, 1993; Weinstein et al., 1997).

Culture-based detection methods, the traditional means of demonstrating microbial viability, tend to be laborious, time consuming and slow to provide results. Since different bacteria need special culture conditions, one of limitations of the traditional culture approach is that not all pathogens are suitable to be cultured.

In our study, the positive rate of traditional culture was only 39.1%, compared with 100% for mNGS. mNGS shows higher sensitivity than traditional methods in pathogen detection of *V. vulnificus*, which is consistent with the results of previous studies (Bonamonte et al., 2013). 5 of 14 patients in this study, blood samples were simultaneously tested for blood culture and mNGS, and the diagnosis was based on the results of mNGS. For these patients, they benefited. For others, since the detection of traditional culture and mNGS is not synchronized, the results may also be biased.

mNGS, as a culture-independent, unbiased, and hypothesis-free approach, has emerged as a diagnostic method for infection in recent years. Infection due to a broad array of organisms can occur in patients with traumatic injury associated with water exposure, we can obtain more meaningful pathogen information than traditional culture methods and PCR. On the other hand, timely empiric anti-infective therapy is very important to save patients with *Vibrio vulnificus* infection. The outcomes of mNGS are less likely influenced by prior antibiotic expose than culture-dependent methods.

In our study, the total cost of culture-positive patients is lower than that of mNGS-positive patients (P=0.099). The disparity is not attributed to the expense of mNGS testing, but rather stems from the necessity for distinct treatment in this particular condition. At present, the high cost is one of the bottlenecks that restrict the widespread of mNGS in clinical practice. A study mentioned that the average cost of mNGS is 3,000 renminbi [RMB] (approximately $400) per specimen in China, which is higher than that for any single traditional pathogenic test (600–700 RMB for culture test, 320 RMB for Cryptococcus antigen test) (Miao et al., 2018). From a health economics perspective, mNGS is able to identify all potential pathogens in a single test, which may be more cost-effective than a series of traditional pathogen screening tests

In terms of etiological detection, we combined mNGS with traditional culture method in this study, and the diagnosis rate was significantly higher than that of traditional culture method alone. Meanwhile, the drug sensitivity test provided a reliable basis for diagnosis and treatment, thus significantly reducing the mortality of patients.

### 4.1 Limitation

As a single-center study, the sample size included was small and the matching could not be completed between the mNGS group and the traditional culture technique. In the following studies, it is expected to further explore the advantages and disadvantages of traditional culture methods and metagenomic sequencing by increasing the number of included cases, so as to obtain more reliable conclusions.

### 4.2 Conclusion

*Vibrio vulnificus* causes rapid disease progression, high medical costs, and may cause disability and death if not diagnosed in time. Unfortunately, the positive rate of traditional culture is not high, despite early diagnosis is very important. For the patients with skin soft tissue infection and necrotizing fasciitis caused by marine wounds, mNGS can be used as an effective way for early pathogen diagnosis, and can make up for the deficiency of traditional culture.

## Data availability statement

The data contains the mNGS results of five patients, the date of patient 5 had lost during the company's data migration. DOI: 10.6084/m9.figshare.24467860.

## Ethics statement

The study involving human participants were reviewed and approved by Institutional Review Board of the Fifth Affiliated Hospital of Sun Yat-sen University (Zhuhai, China). (No. ZDWY [2023] Lunzi No. [K226-1].

## Author contributions

XLiu and JX participated in study design, manuscript writing and editing. XLi and CW participated in data collection and analyses. XLiu, XLi and CW participated in manuscript writing. ZG participated in samples collection and culturing. TX participated in mNGS testing. YM participated in surgical and medical treatments. YJ and ML participated in data collection. All authors contributed to the article and approved the submitted version.
